# Mobile phones to support adherence to antiretroviral therapy: what would it cost the Indian National AIDS Control Programme?

**DOI:** 10.7448/IAS.17.1.19036

**Published:** 2014-09-02

**Authors:** Rashmi Rodrigues, Lennart Bogg, Anita Shet, Dodderi Sunil Kumar, Ayesha De Costa

**Affiliations:** 1 Division of Global Health, Department of Public Health Sciences, 17177 Karolinska Institutet, Stockholm, Sweden; 2 St. John's National Academy of Health Sciences, Bangalore, India; 3 School of Health, Care and Social Welfare, Mälardalen University, Västerås, Sweden; 4 National AIDS Control Organisation, New Delhi, India; 5 Karnataka AIDS Prevention Society, Bangalore, India

**Keywords:** mHealth, mobile phones, adherence reminders, costing, India, National AIDS Control Program

## Abstract

**Introduction:**

Adherence to antiretroviral treatment (ART) is critical to maintaining health and good clinical outcomes in people living with HIV/AIDS. To address poor treatment adherence, low-cost interventions using mobile communication technology are being studied. While there are some studies that show an effect of mobile phone reminders on adherence to ART, none has reported on the costs of such reminders for national AIDS programmes. This paper aims to study the costs of mobile phone reminder strategies (mHealth interventions) to support adherence in the context of India's National AIDS Control Program (NACP).

**Methods:**

The study was undertaken at two tertiary level teaching hospitals that implement the NACP in Karnataka state, South India. Costs for a mobile phone reminder application to support adherence, implemented at these sites (i.e. weekly calls, messages or both) were studied. Costs were collected based on the concept of avoidable costs specific to the application. The costs that were assessed were one-time costs and recurrent costs that included fixed and variable costs. A sequential procedure for costing was used. Costs were calculated at national-programme level, individual ART-centre level and individual patient level from the NACP's perspective. The assessed costs were pooled to obtain an annual cost per patient. The type of application, number of ART centres and number of patients on ART were varied in a sensitivity analysis of costs.

**Results:**

The Indian NACP would incur a cost of between 79 and 110 INR (USD 1.27–1.77) per patient per year, based on the type of reminder, the number of patients on ART and the number of functioning ART centres. The total programme costs for a scale-up of the mHealth intervention to reach the one million patients expected to be on treatment by 2017 is estimated to be 0.36% of the total five-year national-programme budget.

**Conclusions:**

The cost of the mHealth intervention for ART-adherence support in the context of the Indian NACP is low and is facilitated by the low cost of mobile communication in the country. Extending the use of mobile communication applications beyond adherence support under the national programme could be done relatively inexpensively.

## Introduction

Adherence to antiretroviral treatment (ART) is critical for ensuring good clinical outcomes in people living with HIV/AIDS (PLHIV) [1, 2]. This is of importance to patients and health systems alike. To improve treatment adherence in PLHIV receiving treatment, healthcare systems have attempted to invest in low-cost interventions that could improve ART adherence. Given the ubiquity of mobile phones (6.8 billion estimated mobile phone subscriptions globally [3]), mobile phone reminders for the improvement of treatment adherence in chronic disease, especially HIV infection, have been increasingly explored [4–6].

India is home to 2 million of the 33 million PLHIV globally with a national HIV-infection prevalence of 0.3% [7, 8]. To provide treatment and follow-up services to these individuals, the government of India implements the National AIDS Control Program (NACP). The NACP provides care, treatment and support to PLHIV (free of cost to the patient) through ART centres across the country. Each centre has a team that includes a medical officer, a nurse, a counsellor, a pharmacist, a data-entry operator and an outreach worker. Counsellors at ART centres identify barriers to adherence and provide adherence counselling to patients on treatment. As of December 2012, 570,620 infected individuals had been started on ART at 380 ART centres in the country. Second-line ART for those failing first-line ART is provided only to those who have been on first-line ART for at least six months at a public ART centre or public–private partnership facility [7]. Providing second-line ART is six times more expensive than first-line ART [9]. This stresses the need to ensure optimal adherence to first-line ART from an individual and health-system perspective [10].

Mobile phone applications that support treatment adherence address forgetfulness; an important barrier to treatment adherence, even in the Indian context [7, 8, 11]. Further, mobile phone reminders that target forgetfulness to improve ART adherence have shown mixed results globally [6, 12, 13]. Improved ART adherence with weekly short messaging-service (SMS) reminders has been reported in two studies from Kenya [1, 5, 14]. A similar outcome was seen in a pilot study using an mHealth intervention that included both SMS reminders and interactive voice-response (IVR) calls in our setting [15], following which, we began to test this mHealth intervention in a randomised controlled trial in South India [16]. In contrast, SMS reminders had no effect on ART adherence in Cameroon [17]. However, a pooled analysis of the results from the Kenyan and Cameroon studies suggests the overall effectiveness of SMS reminders on ART adherence [18].

While most published studies focus on the effects of the intervention, few have obtained and analysed primary cost data and estimated mHealth intervention costs at scale [19, 20]. In this paper, we present the costs that the NACP in India would incur to deploy mHealth interventions on a national scale for ART-adherence support.

## Methodology

### Study setting

The study setting included two tertiary level hospitals that implement the NACP through ART centres on their premises. St. John's Medical College Hospital, a private non-profit teaching hospital, in Bangalore, Karnataka, implements the NACP through a public–private partnership model. The ART centre at this hospital is responsible for the treatment and follow-up of approximately 1,500 PLHIV enrolled under the NACP. The other was a public tertiary care teaching hospital in Mysore, also in Karnataka. This hospital manages the treatment and follow-up of 2,500 PLHIV under the NACP.

Karnataka with a population of 61 million and an HIV-prevalence rate of 0.63% is one of four HIV high-prevalence States in India [21]. As of December 2012, the State had 90,000 PLHIV on treatment at 44 ART centres [7].

### The mHealth intervention for adherence support

The mHealth intervention that has been costed in this paper was developed for use in the HIVIND trial. The HIVIND trial [16] is a randomised controlled trial to test the effect of an mHealth intervention on adherence to antiretroviral therapy in PLHIV in South India. The intervention, prior to deployment in the trial, was pilot tested in a cohort of PLHIV on treatment and showed an improvement in adherence [15].

The mHealth intervention deployed in the HIVIND trial had two components; the first was an automated IVR call and the second was a passive, neutral, picture message (SMS). Every patient in the intervention arm of the trial received each component of the mHealth intervention once a week. Details of the intervention are reported in the HIVIND trial protocol [16].

### Assumptions

The mHealth intervention that has been costed in this paper is from the HIVIND trial. Hence, costs that were not considered included the additional costs incurred to follow the trial protocol and maintain the research quality in the trial context. These costs would not be incurred in a routine programme setting. In view of the relatively limited time period used for estimating costs, discounting was not applied on costs or benefits.

We considered the mobile-solutions agency that developed and managed the mHealth intervention for the trial to be the most economical as they offered the lowest price through a competitive bidding process for developing and deploying the intervention. In the HIVIND trial with 631 patients, 316 received the mHealth intervention. Further, each 30-second IVR call was priced at 1.50 Indian Rupees (INR), while the SMS was priced at 0.20 INR. If the same intervention were implemented at scale, the prices would decrease to 0.24 INR per 30-second IVR call and 0.19 INR per SMS if the number of calls and SMSs were to exceed 500,000, that is, when patient numbers exceed 10,000.

Costs for the mHealth intervention were studied from the perspective of the Indian NACP. Costs were collected based on the concept of avoidable costs specific to the mHealth intervention. The concept of avoidable costs refers only to the inclusion of costs that are contingent on the mHealth intervention, and all other costs were considered as sunk costs; that is, costs incurred even if the intervention was not undertaken. Sunk costs (e.g. costs of buildings) were not included in the study. The costs that were assessed were one-time costs as well as recurrent costs (the latter included fixed and variable costs). In addition, Appendix A describes the actual cost calculations for the HIVIND trial.

A sequential costing procedure was used. It involved first identifying the resource used in natural units (minutes of staff time and/or other relevant units), measuring the resource used and subsequently pricing the resource using standardized prices.

### Costs considered

#### One-time costs

Costs that are incurred only once during the entire programme (i.e. one cycle of the programme =5 years), primarily mHealth intervention–development costs.

#### Recurrent costs

These are costs incurred every year. They include fixed costs and variable costs. Fixed costs do not vary with a change in the amount or quantity of an output or service (e.g. salaries of staff that are not dependent on the number of patients seen). Variable costs are costs that are dependent on the quantity of output (e.g. the number of beneficiaries of a service).

The costs studied were stratified at three different levels in the deployment of the mHealth intervention: (1) national-programme level (2) ART-centre level and (3) patient level.

#### National-programme level

##### One-time costs

These were costs incurred for the development of the mHealth intervention, costs for recording the mHealth intervention in different languages (each language in which the reminder was recorded incurred a cost of 2,000 INR) and costs for the development of the web interface (this is the portal that captured reports of the mHealth intervention delivery and receipt). The web interface facilitated the technical monitoring of the mHealth intervention. The above costs were those paid to the mobile-solution agency in the HIVIND trial. Costs for equipment consisting of a laptop and a mobile phone used for monitoring the mHealth intervention and communicating with other ART centres deploying them were also considered under one-time costs.

##### Recurrent fixed costs

These included the costs for the maintenance of the hardware and the development and maintenance of the web interface. A proportion of the salary for the programme officer responsible for the nationwide deployment of the mHealth intervention through the NACP was also included. Overheads at the programme level were calculated as a proportion of fixed costs.

#### ART-centre level

##### One-time costs

These included the costs of equipment (i.e. one mobile phone and one laptop used for demonstrating the mHealth intervention to the patient). These one-time costs were calculated based on costs incurred at the ART centre, St. John's Medical College Hospital.

##### Recurrent fixed costs

These were the fixed costs incurred by the ART centre, St. John's Medical College Hospital and included the staff costs: (1) 10% annual time of a centre functionary, that is, an ART medical officer responsible for the overall monitoring of the mHealth interventions; (2) 10% annual time of the data manager responsible for updating patient information, the follow-up of patient responses captured by the web interface and identifying and reporting issues that arose with the functioning of the mHealth intervention; and (3) overheads that were a proportion of fixed costs incurred at the ART-centre level.

It should be noted that fixed costs are dependent on the scale of the intervention. When the intervention is scaled up to the national level, the recurrent fixed costs at ART-centre level will increase step-wise and will gradually change into recurrent variable costs.

#### Patient level

##### Recurrent variable costs

These included costs per patient for the mHealth intervention (IVR calls and SMSs). These variable costs change based on the number of patients when the application is scaled up. The time spent by the ART-centre counsellor (i.e. one hour per patient per year) on the following activities was considered: (1) training patients to receive and respond to the mHealth intervention, (2) follow-up of mHealth intervention receipt with the patient, and (3) routine patient counselling for adherence at monthly follow-up visits at the ART centre. To obtain these costs, the counsellors in the HIVIND trial maintained diaries for the time spent with each patient on these tasks.

##### Sensitivity analysis

Sensitivity analysis was carried out by varying (1) the number of patients and the number of ART centres (i.e. the costs for scale-up); and (2) the type of mHealth intervention deployed, that is, (1) only IVR, (2) only SMS or (3) both SMS and IVR used in combination.

##### Costs for the scale-up

For the scale-up of the intervention, the presence of 22 official regional languages recognised by the 7th Schedule of the Indian Constitution, and English were considered. Different estimates for the numbers of ART centres and PLHIV on treatment were considered. The numbers of ART centres were arrived at from NACP reports of the existing number of ART centres and projected estimates of the number of ART centres for 2017 [7, 22].

Three different estimates of expected PLHIV numbers under India's NACP in 2017 were also considered: (1) a published mathematical model, which estimated that there would be 500,000 patients on ART in 2017 [23]; (2) the Planning Commission, government of India estimate of 800,000 patients on ART by 2017 [24]; and (3) the National AIDS Control Organisation (NACO) estimates for 1,000,000 patients on ART by 2017 [7, 22].

##### Total costs per year for deployment of the mHealth intervention (total intervention cost)

These were calculated as a function of fixed and variable costs using the formula: total cost for deployment of the IVR call plus SMS=programme-level cost+number of centres×centre-level cost+n×patient-level cost; where n=the number of patients.

The total costs for each component of the intervention were calculated using the same formula; however, the costs that were contributed by only that component of the intervention were considered (i.e. IVR or SMS).

##### Costs per patient per year

These were calculated by dividing the total costs per year for deployment of the mHealth intervention by the number of patients expected to receive the intervention during that year.

### Ethics statement

The study was part of the HIVIND trial. Ethical clearance for the study was obtained from the Institutional Ethics Committee, St. John's Medical College.

## Results

### Overall costs for co-ordination and implementation of the intervention at each level

A cost of 371,690 INR/year was estimated at national-programme level for the overall co-ordination and monitoring of the intervention, as can be seen in the first section of Table 1. Similarly, a total cost of 54,450 INR/year was estimated at ART-centre level (per ART centre), as can be seen in the second section of Table 1.

**Table 1:** Costs for coordinating the overall implementation and monitoring of the mHealth intervention to support adherence to first-line ART at (a) national-programme level (b) ART-centre level

At national-programme level	Actual cost	Cost/year (INR)
Annualised one-time costs		
Equipment (one laptop and one mobile phone)(total cost approximately 40,000 INR)^a^	40,000 INR	8,000.00
Recording the IVR call (cost/language=2,000 INR×23 languages)^b^	46,000 INR	9,200.00
Development of the web interface to follow up functioning and capture patient responses (cost=10,000 INR, as quoted by the mobile-solution agency)^a^	10,000 INR	2,000.00
Equipment maintenance (cost=1,400 INR for 2 years)	1,400 INR for 2 years	700.00
Recurrent fixed costs		
mHealth intervention service maintenance (based on costs provided by the mobile-solution agency)	1,500/month	18,000.00
Project manager to oversee functioning and co-ordinate the mHealth intervention (100% annual time)	25,000/month salary	300,000.00
Overheads 10% of fixed costs	Based on overheads	33,790.00
Total health-system (programme-level) cost		371,690.00 (5999.83 USD)
**ART-centre level**		
One-time costs		
Equipment (one laptop and one mobile phone)(total cost approximately 40,000 INR)^a^ for managing patient information and demonstration of the mHealth intervention to the patient	40,000 INR	8,000.00
Fixed costs		
Equipment maintenance (cost=1,400 INR for 2 years based on the HIVIND trial)	1,400 INR for 2 years	700.00
ART medical officer (10% of 312,000 INR/year, i.e. annual salary)	10% of 312,000	31,200.00
Data manager (10% of 96,000 INR/year, i.e. annual salary)	96,000 INR/year	9,600.00
Overheads 10% of fixed costs		4,950.00
Total costs per centre		54,450.00 (878.93 USD)

a The NACP runs on a five-year cycle; hence, actual costs have been divided by 5 to obtain annualised costs;

b http://lawmin.nic.in/coi/EIGHTH-SCHEDULE.pdf.

1 USD=61.95 INR as of 26 December 2013.

Table 2 describes the costs for the mHealth intervention per patient per year in the trial and the costs if the intervention was to be implemented at scale. A variable cost of 126.40 INR (per patient per year) was estimated at patient level for the mHealth intervention comprising of the weekly IVR call and the SMS reminder, as seen in Table 2. The variable cost per patient per year for IVR calls only was 116.40 INR and the variable cost for the SMS only was 48.40 INR/patient/year. At scale, if total patient numbers exceed 10,000, the variable costs for the mHealth intervention (both elements) at the level of the patient decrease to 60.36 INR/patient/year, the variable costs for only the IVR decrease to 50.48 INR/patient/year and the variable costs for the SMS decrease to 47.88 INR/patient/year.

**Table 2:** Variable costs for the mHealth intervention per patient per year in the trial and at scale

Costs per patient in the trial		
IVR call/patient (cost/30 sec call=1.50 INR, as quoted by the mobile-solution agency for the trial)	1.50 INR/week×52 weeks	78.00
SMS reminder/patient (cost of 1 SMS reminder=0.2 INR, as quoted by the mobile-solution agency for the trial)	0.20 INR/week×52 weeks	10.40
Counsellor^a^ (1 hour/patient/year, salary=96,000 INR/year)	96,000 INR/year	38.00
Variable cost per patient for the (IVR call+SMS reminder)/year in the trial		126.4 (2.04 USD)
Variable cost per patient for the IVR call only/year in the trial		116.00 (1.87 USD)
Variable cost per patient for the SMS reminder only/year in the trial		48.40 (0.78 USD)
Costs per patient if total patients >10,000		
IVR call/patient (cost/30 sec call=0.24 INR, as quoted by the mobile-solution agency)	0.24 INR/week×52 weeks	12.48
SMS reminder/patient (cost of 1 SMS reminder=0.19 INR, as quoted by the mobile-solution agency)	0.19 INR/week×52 weeks	9.88
Counsellor^a^ (1 hour/patient/year, Salary=96,000 INR/year)	96,000 INR/year	38.00
Variable cost per patient for the (IVR call+SMS reminder)/year		60.36 (0.97 USD)
Variable cost per patient for the IVR call only/year		50.48 (0.81 USD)
Variable cost per patient for the SMS reminder only/year		47.88 (0.77 USD)

a Counsellor diaries indicated that the counsellors spent about 5 minutes/patient/every month to discuss the application. The counsellors’ time for the mHealth intervention therefore amounted to 60 min/patient/year (Salary: 8,000 INR/month); 1 USD=61.95 INR as of 26 December 2013.

### Sensitivity analysis

The estimates shown in Tables 1 and 2 were then used in the sensitivity analysis, where the costs per patient for varying numbers of enrolled patients, the number of ART centres and the type of intervention were estimated (Table 3). The costs for deploying the mHealth intervention vary because of the assumptions regarding the number of patients; the costs of services are lower with increasing patient numbers as the fixed costs are divided by a larger number of patients.

**Table 3:** Sensitivity analysis of costs of the mHealth intervention for ART adherence support based on the number of patients and type of mHealth intervention

All costs in INR	Cost/year, 2012 (INR)		Cost/year (INR) 2017	
Number of ART centres	380 [7]	450^a^	450 [24]	600 [7, 22]
Number of languages^b^	23	23	23	23
Number of patients (n)	600,000 [7]^c^	500,000 [23]	800,000 [24]	1,000,000 [7, 22]
Costs for intervention development based on languages included, i.e. (23 languages×2,000 INR per language/5) where 5 is the number of years one NACP cycle lasts	9,200	9,200	9,200	9,200
Co-ordination cost based on the number of ART centres^d^ (i.e. ART center level cost from Tables 1×number of ART centres)	20,691,000	24,502,500	24,502,500	32,670,000
Total intervention cost (national programme-level cost+number of centres×centre-level cost+n×patient-level cost)	57,278,690	55,054,190	73,162,190	93,401,690
Intervention cost/patient/year=cost of (IVR+SMS reminder)/n	96.46 (1.55 USD)	110.11 (1.77 USD)	91.45 (1.47 USD)	93.40 (1.50 USD)
Total cost of only the IVR call (national programme-level cost+number of centres×centre-level cost+n×patient-level cost for IVR only)	51,350,690	50,114,190	65,258,190	83,521,690
Cost of IVR call/patient/year=cost of IVR only/n	85.58 (1.38 USD)	100.23 (1.61 USD)	81.57 (1.31 USD)	83.52 (1.34 USD)
Total cost of only the SMS reminder (national programme-level cost+number of centres×centre-level cost+n×patient-level cost for SMS reminder only)	49,790,690	48,814,190	63,178,190	80,921,690
Cost of the SMS reminder/person/year=cost of SMS reminder only/n	82.98 (1.33 USD)	97.63 (1.57 USD)	78.97 (1.27 USD)	80.92 (1.30 USD)

a Based on the existing 380 centres currently in existence and that one of the estimates by NACO indicates an expansion to 450 centres in 2017, we therefore assumed 450 ART centers for 500,000 patients in 2017;

b http://lawmin.nic.in/coi/EIGHTH-SCHEDULE.pdf, the 23 languages include 22 Indian languages and English;

c The actual number of patients on ART in December 2012 was 570,620, for the purpose of cost calculations this figure has been rounded up to 600,000 in 2012; 1 USD=61.95 INR as of 26 December 2013; The reference years for costing were 2012 and 2017.

d Centre-level costs were multiplied by the number of ART centres (1) existing in 2012 and (2) expected in 2017. The number of ART centres used in the scale-up was based on the National AIDS Control Organisation estimates [7, 22]. NACO estimates that 450 ART centres are required for 800,000 patients and 600 ART centres are required for 1,000,000 patients.

In Table 3, the co-ordination cost (20,691,000 INR), based on the number of ART centres, was arrived at, by multiplying the number of ART centres (380) with the co-ordination cost per centre (54,450 INR, as shown in Table 1). The total intervention cost (57,278,690 INR) was arrived at by adding the total programme-level cost (371,690 INR, as shown in Table 1), the co-ordination costs based on the ART centers (20,691,000 INR) and the total variable cost per patient (116.40 INR, as shown in Table 1), multiplied by the number of patients (i.e. 600,000 in 2012). The total intervention cost per patient per year (96.46 INR) was arrived at by dividing the total intervention cost (57,278,690 INR in 2012) by the number of patients (600,000 in 2012). Similar calculations were made with different assumptions for the number of patients and the number of ART centres (columns 2–4 in Table 3). Separate costs based on the type of intervention (i.e. IVR alone or SMS alone) are shown in the last four rows of Table 3. The intervention cost per patient per year ranges from 91.45–110.11 INR for the IVR and SMS combined, 81.57–100.33 INR for the IVR alone and 78.97–97.63 INR for the SMS alone based on the number of patients and ART centres deploying the intervention.

## Discussion

Our estimates for the deployment of the mHealth on a national scale range from 79 to 110 INR/patient/year (i.e. approximately 1.27–1.77 USD) based on the type of intervention; that is, SMS only, IVR call only, IVR call plus SMS, the number of patients and the number of ART centres considered at scale. The low costs incurred in our study were due to the Asterisk telephony software and Linux operating system used for the deployment of the mHealth intervention, both of which did not require licences to be purchased. In addition, the low cost of mobile telephone services in our setting in South India made the mobile phone application in our study less expensive, a situation that may be similar in other low- and middle-income (LMIC) settings, but which might differ from high-income settings. At 0.01 USD/minute, the Indian telecom industry provides the lowest mobile phone calling rates globally [25].

Differing estimates suggest that between 500,000 to 1,000,000 patients are expected to receive first-line antiretroviral therapy during the NACP phase IV (2012–2017) [7]. While this number varies significantly based on the source of information used, it is likely that the numbers will be close to a million, given the recent WHO recommendations to start ART at higher CD4 cell levels than currently used in the programme [26]. The NACP is likely to adopt the new WHO recommendations, significantly increasing the number of patients eligible for first-line ART.

The proposed budget for the NACP IV is approximately 128,240 million INR for five years [27]. Based on our assessment of cost, the total programme costs for a scale-up of the mHealth intervention application for 1,000,000 patients expected to be on treatment by 2017 is estimated to be 0.36% of the total five-year NACP IV budget.

First-line ART costs the Indian NACP approximately 5,000 INR/patient/year, while second-line ART costs 30,000 INR/patient/year [7]. Approximately 600,000 PLHIV are currently on first-line ART [7]. The NACP expects 2–3% of those on ART to fail treatment [7]; that is, approximately 30,000 infected individuals are likely to require second-line ART in 2017. This expenditure could increase in the future with a growing number of PLHIV starting first-line ART and with an increasing cumulative number of ART failures requiring second-line ART. Studies in low- and middle-income settings indicate that good adherence could provide substantial economic benefits to healthcare systems by limiting the necessity for second-line ART regimens [28–30]. Further, good adherence to ART has the potential to reduce hospital admissions, shorten hospital stays and lower hospitalisation costs [31]. An intervention involving peer health workers in Uganda that included an mHealth component was considered to be cost saving if it prevented an average of 1.5 patients per year from switching to second-line ART [19]. Low-cost applications targeting adherence could improve adherence and minimise hospitalisation costs for the NACP.

While mHealth interventions are attractive for adherence support, they hold the potential for disclosure of HIV status if the mHealth intervention is intercepted by others [12]. In addition, the popularity of the IVR call over the SMS in our context [15, 32] needs consideration. The costs of the mHealth intervention that is designed will vary based on these considerations.

A previous study in our setting in the year 2009 reported a mobile phone ownership of 73%; ownership was associated with being male, literate and employed [32]. This merits consideration, as the lack of mobile phones would be an obstacle to delivering the reminder to approximately 25% of PLHIV without a mobile phone, a significant proportion of whom are likely to be female. However, given that mobile phone ownership has been increasing rapidly since that time, especially due to the decreasing costs of handsets and the low cost of mobile communication in the Indian context, it is likely that most PLHIV will have access to a mobile phone.

### Other potential uses of mobile phone technology in healthcare

A vast, rapidly growing body of literature, describes the use of mHealth technology to support various facets of prevention and management of HIV infection in LMIC settings. While the most popular application of mHealth is for adherence support, the technology has also been successfully used to schedule hospital appointments and obtain diagnostic reports [33–35]. mHealth is also being explored to ensure the retention of HIV patients in healthcare [36]. Further, behaviour-change education for preventing HIV infection [37] and partner testing to reduce the risk of HIV transmission have been promoted with mobile phones [38, 39]. More recent innovations involve performing laboratory investigations using mobile devices [40]. Our cost data could also be projected to the expected expenditures for some of these mHealth services if these are taken up for implementation in the country.

### Methodological issues

Costs for the application could change based on the changes in currency exchange rates. Costs may differ depending on the provider of the technology (i.e. the technical-solution agency hired to develop and deploy the mHealth intervention). Additionally, the costs of IVR calls and SMSs may change over time.

Costs to other national programmes will vary based on the costs of mobile phone services in those settings, as well as programme costs, staff costs and so on; therefore, the findings from our study are relevant only in the context of the Indian national programme.

## Conclusions

The ubiquity and low cost of mobile phones in India make mobile phone technology a potential tool to support treatment adherence and other aspects of HIV-related care in the Indian context. Previous studies in our setting indicated a preference for IVR calls for ART-adherence support and showed an improvement in ART adherence in PLHIV on treatment. Estimating the costs of the mHealth intervention was considered essential prior to deploying it at scale, especially as no prior estimates of such costs exist in the Indian context. The costs estimated in our study indicated that the cost of mHealth interventions for ART-adherence support in the context of the Indian NACP is low, regardless of whether calls, messages or both modalities are used. The study also provides some indication of programme costs for other HIV healthcare-related functions that the mobile phone could potentially be used to support. Given that there have been a number of pilot projects and trials in the area of mHealth and HIV/AIDS for some years now, there are increasing calls to scale up and integrate these projects into national HIV programmes.

## Appendix A

### Costs in the trial

The total cost per patient was calculated as TC/n=(TFC+ n×VC)/n, where TC=total cost, n=number of participants, TFC=total fixed costs and VC=variable cost per patient.

TFC=371,690+54,450=426,140, TC=426,140+316×126.4=466,082.40.

**TC/n=466,082.40/316=1474.94 INR (23.81 USD) per patient per year**

TC/n IVR=(426,140+316×116.40)/316=1464.94 INR (23.65 USD) per patient per year

TC/n SMS=(426,140+316×48.40)/316=1396.94 INR (22.54 USD) per patient per year
